# Limited association between serum vancomycin pharmacokinetic/ pharmacodynamic indices and clinical outcomes in gram-positive complicated urinary tract infections

**DOI:** 10.3389/fphar.2026.1772367

**Published:** 2026-07-16

**Authors:** Yaxin Fan, Jinjin Zhao, Yuancheng Chen, Lin Xi, Hailan Wu, Beining Guo, Nanyang Li, Ping Yang, Qiyu Bian, Jufang Wu, Jing Zhang

**Affiliations:** 1 Institute of Antibiotics, Huashan Hospital, Fudan University, Shanghai, China; 2 Key Laboratory of Clinical Pharmacology of Antibiotics, National Health Commission of People’s Republic of China, Shanghai, China; 3 National Clinical Research Center for Aging and Medicine, Huashan Hospital, Fudan University, Shanghai, China; 4 Clinical Pharmacology Research Center, Huashan Hospital, Fudan University, Shanghai, China; 5 Joint Laboratory of Hospital and Enterprise for Pathogen Diagnosis of Drug-resistant Bacterial Infections and Innovative Drug R&D, Shanghai, China

**Keywords:** complicated urinary tract infection, pharmacokinetic/pharmacodynamic, serum therapeutic drug monitoring, trough concentration, vancomycin

## Abstract

**Background:**

Vancomycin, primarily excreted through the urine, is used for complicated urinary tract infections (cUTIs) caused by Gram-positive bacteria. Although serum therapeutic drug monitoring (TDM) is usually performed in vancomycin therapy, its benefits and risk factors in patients with cUTIs remain unclear.

**Methods:**

Adults with Gram-positive bacterial cUTIs receiving serum vancomycin TDM were enrolled from three prospective, multicenter trials. Minimal inhibitory concentration (MIC) was measured by agar dilution for pathogens collected from all patients. Clinical characteristics and pharmacokinetic/pharmacodynamic (PK/PD) indices were analyzed between the vancomycin treatment success and failure groups.

**Results:**

A total of 74 adult patients with cUTIs were enrolled. Median initial daily dose of vancomycin was 1.0 g (interquartile range [IQR], 1.0–2.0 g), given in divided doses every 12 h or as a once-daily regimen. Most concomitant antibiotics targeted Gram-negative bacteria or fungi, with very limited anti-Gram-positive co-therapy. The median serum trough concentration (C_min_) of vancomycin was 9.22 mg/L (IQR, 4.36–14.33 mg/L) and 24-h area under the concentration-time curve to MIC (AUC_24_/MIC) was 455 (IQR, 268–627). Despite low attainment of the AUC_24_/MIC 400–600 target (27/74, 36.5%), the treatment success rate was 86.5% (64/74) and the nephrotoxicity rate was 4.1% (3/74). Urinary pathogens isolated included *Enterococcus* spp (55/74), *Streptococcus* spp (10/74), and *Staphylococcus aureus* (9/74), including eight methicillin-resistant *S*. *aureus* [MRSA]). Both solid tumor and *S. aureus* infection showed exploratory associations with vancomycin treatment failure. Patients with solid tumor had a lower probability of attaining the target AUC_24_/MIC, likely due to the elevated MIC of the predominant *Enterococcus* strains in this population. In contrast, patients with *S. aureus*-induced cUTIs achieved higher AUC_24_/MIC levels. All isolates exhibited low MICs (≤1 mg/L), no heteroresistance was detected, and the predominant molecular type was clone complex 5 (CC5).

**Conclusion:**

Vancomycin was effective in *Enterococcus*-dominant cUTIs and had a modest response in a few MRSA cases, despite low PK/PD target attainment. Serum C_min_ and AUC_24_/MIC showed limited association with clinical outcomes, whereas solid tumor and *S. aureus* infection showed exploratory associations with treatment failure. However, TDM remains valuable for safety monitoring and individualized dosing. These findings should be validated in larger cohorts.

## 1 Introduction

Complicated urinary tract infections (cUTIs) are among the most common infectious diseases, giving rise to major clinical concern and economic burden (Foxman, 2014). Gram-positive uropathogens such as *Enterococcus* spp., *Staphylococcus* spp. and *Streptococcus* spp., are more likely implicated in cUTIs than in uncomplicated UTIs (Flores-Mireles et al., 2015). Vancomycin, mainly excreted via the kidneys in urine, attains high urinary drug concentrations. It is recommended for cUTIs caused by methicillin-resistant *S. aureus* (MRSA) strains and serves as an alternative for *Enterococcus*-induced cUTIs (Rando et al., 2022).

Vancomycin has been in clinical use for over 60 years, but the optimal dosing strategy remains controversial. Clinicians have always attempted to optimise vancomycin dosing to minimise toxicity and improve efficacy. The 24-h area under the concentration-time curve to minimal inhibitory concentration (AUC_24_/MIC) ratio was the key pharmacokinetic/pharmacodynamic (PK/PD) index predictive of efficacy. The AUC_24_/MIC targets are updated from >400 to 400–600 or 400–650, but this PK/PD target was primarily derived from patients with MRSA bloodstream infections (Rybak et al., 2020). The 2020 Chinese guideline still recommends the trough concentration (C_min_) 10–15 mg/L as a therapeutic drug monitoring (TDM) target for general infections (He et al., 2020). Nonetheless, vancomycin TDM is usually based on serum C_min_ in clinical practice. Based on these targets, insufficient data are available for vancomycin treatment in other Gram-positive infections such as the urinary tract infections (Rybak et al., 2009; Ye et al., 2016; He et al., 2020; Rybak et al., 2020; Matsumoto et al., 2022). However, it remains unclear whether vancomycin serum TDM is beneficial for patients with cUTIs caused by Gram-positive bacteria, and whether the recommended vancomycin serum C_min_ or PK/PD targets are appropriate for this specific indication.

Therefore, this study aimed to evaluate the role of serum vancomycin TDM in patients with cUTIs. We also sought to identify patient subgroups at high risk for treatment failure who might derive the benefit from serum TDM.

## 2 Methods

### 2.1 Study design

Data on patients with cUTIs included in this study came from three prospective, multicenter, observational studies that included patients with Gram-positive bacterial infections and were conducted from September 2012 to December 2023 in 26 hospitals across China. The studies were approved by the Ethics Committee of Huashan Hospital of Fudan University and were conducted in accordance with the principles of the Declaration of Helsinki and Good Clinical Practice Guidelines. Written informed consent was obtained from all the patients, and all consented voluntarily. The three studies were registered with the Chinese Clinical Trial Registry (www.chictr.org.cn), accession number: ChiCTR- OPC-16007920 (14 February 2016)/ChiCTR-OPC-17012567 (4 September 2017)/ChiCTR2100046718 (17 May 2021). Patients diagnosed with cUTIs were included in this study.

### 2.2 Study population

All enrolled patients were adults (≥18 years old) with cUTIs caused by Gram-positive bacteria, such as MRSA, *Enterococcus*, and *Streptococcus*. All enrolled patients had microbiologically confirmed Gram-positive cUTIs, and vancomycin was initiated at the physician’s discretion (empirically or after pathogen confirmation) without delaying therapy for susceptibility testing. Additionally, patients with cUTIs caused by Gram-positive bacteria who were allergic to β-lactam antibiotics were also included. The diagnosis was based on both clinical evidence (including symptoms, signs, and laboratory tests) and microbiological evidence (from urine cultures). The detailed cUTIs diagnostic criteria were as follows: (I) including at least one of the following comorbid factors: indwelling catheter, stent or intermittent bladder catheterization; post-void residual urine >100 mL; obstructive urinary tract disease, such as bladder outlet obstruction, neurogenic bladder, stones and tumors, etc.; vesicoureteral reflux or other dysfunction; urinary diversion; urothelium injury from chemotherapy or radiation therapy; postoperative urinary tract infection; renal insufficiency, renal transplant, urinary retention due to benign prostatic hypertrophy; immunodeficiency; (II) white blood cells ≥10/mm^3^ in non-centrifuged urine or >5/high power field (HPF) in centrifuged urine; (III) Positive urine culture ≥100,000 colony forming units (CFU)/mL from clean-catch, midstream urine. Patients were excluded if they received antimicrobial therapy other than vancomycin effective for Gram-positive bacteria for more than 24 h within 72 h before enrollment. Prior vancomycin exposure before enrollment was allowed and not excluded.

### 2.3 Vancomycin administration and sampling

The initial dose of vancomycin was determined based on the recommendations in the package insert and clinical guidelines. The individual dosing regimen was adjusted according to the results of TDM. Serum samples were collected from patients with normal renal function pre-dose (within 0.5 h) to determine the C_min_, and 0.5–1-h post-dose to determine the peak concentration (C_max_) after the fourth dose. In case of glomerular filtration rate <30 mL/min using CKD-EPI creatinine equation (Inker et al., 2012) or renal replacement therapy, serum samples were taken at the second dose. Vancomycin TDM samples (C_min_, C_max_) were assayed by fluorescence polarization immunoassay (FPIA) or chemiluminescence immunoassay (CMIA) with a detection level of 3.00–100 mg/L. Both methods are commercial immunoassays. Validation was performed using spiked samples at eight concentrations (3, 3.5, 7, 25, 35, 50, 75, and 90 mg/L) with 10 replicates per concentration, and the deviation between measured and spiked concentrations was within 15.0%. Compared with the ultra-performance liquid chromatography reference method (Cao et al., 2014), the correlation equations were y = 0.988x + 0.255 (FPIA) and y = 1.049x – 0.008 (CMIA). The methods were also cross-validated using quality control and clinical patient samples before method transition.

### 2.4 Microbiological data and PK/PD analysis

Clinical isolates of Gram-positive uropathogens were collected from all patients. Vancomycin MIC was verified with the agar dilution method (concentrations: 0.125, 0.25, 0.5, 1, 2, 4, 8, 16 mg/L). The MIC data were interpreted according to the breakpoints in the Clinical Laboratory Standards Institute documents M100 (Institute, 2023). For each species, the frequency distribution of MIC values was calculated, and the MIC inhibiting 50% and 90% of isolates (MIC_50_ and MIC_90_, respectively) were derived from cumulative percentages.

One-compartment model was used to simulate the drug concentration-time curve of each patient with WinNonlin software version 8.3 (Certara, Princeton, NJ, United States) (Fan et al., 2022). AUC_24_ was calculated with MATLAB software version 7.0.1 (MathWorks Inc., Natick, MA) for each dosing regimen, and corrected in case of longer dosing interval (>24 h). MATLAB was used because it supports batch processing of all patients *via* a custom script and allows standardized correction of AUC for dosing intervals longer than 24 h. For dosing intervals longer than 24 h (e.g., q48 h), the AUC was corrected to a standardized 24-h value by averaging the daily AUCs across the extended interval.

### 2.5 Safety and efficacy analysis

Safety analysis was based on safety set, which included the patients who received at least one dose of vancomycin and had valid safety data. Adverse events monitored included nephrotoxicity, ototoxicity, rash, and leukopenia. Nephrotoxicity was the primary safety outcome due to its higher clinical relevance and occurrence in this cohort. Vancomycin-related nephrotoxicity indicates the occurrence of acute kidney injury (AKI). According to the KDIGO criteria (Khwaja, 2012), AKI includes any of the following conditions: (a) an increase in serum creatinine (SCr) by ≥ 26.5 μmol/L within 48 h; (b) a known or presumed increase in SCr by 1.5 times the baseline value within the past 7 days; (c) urine output less than 0.5 mL/kg/h within 6–12 h. Causality was assessed using a five-level scale (definite, probable, possible, unlikely, unrelated). Cases graded as definite, probable, or possible were considered vancomycin-related (see in Supplementary Material). Other potential causes of AKI (e.g., other nephrotoxic drugs) were excluded by the clinical team before assigning causality.

Both clinical efficacy and microbiological eradication were considered in assessment of vancomycin treatment outcome. All patients underwent a follow-up urine culture after completion of vancomycin therapy. Treatment success was defined as eradication of the baseline uropathogens and not requiring additional anti-microbial agent for Gram-positive bacteria within 7 days after the end of vancomycin treatment. Treatment failure was defined as a lack of improvement based on clinical symptoms, signs, and laboratory results after vancomycin treatment, and/or persistent presence of the baseline uropathogens.

### 2.6 Statistical analysis

Statistical analyses were performed with SPSS statistics version 22.0 (SPSS Inc., Chicago, IL, United States). All study variables were summarized with descriptive statistics. The median and interquartile range (IQR) was calculated for continuous variables. Counts and percentages were used to summarize categorical data. The Mann-Whitney U test was used for continuous variables, and Fisher’s exact test for categorical variables. We assessed the associations between treatment outcomes and patient demographics, clinical characteristics, vancomycin concentrations, and AUC_24_/MIC values. Variables with a *P* value <0.1 in the univariate analysis were included in the multivariate analysis. Multivariate logistic regression with backward stepwise selection was performed to identify independent predictors of clinical outcome. *P* < 0.05 was considered statistically significant.

## 3 Results

### 3.1 Clinical characteristics

**FIGURE 1:**
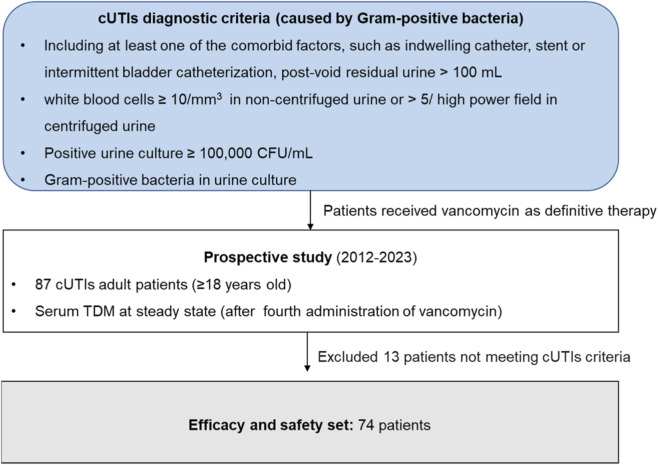
Flowchart for patient enrolment and evaluation in this study cUTIs: complicated urinary tract infections; CFU, colony forming units; TDM, therapeutic drug monitoring.

We respectively conducted data from three prospective, multicenter clinical studies (Figure 1) to preliminarily explore the clinical characteristics of adult patients with confirmed cUTIs caused by Gram-positive bacteria and to determine whether serum TDM is beneficial.

Of the 87 enrolled adult patients, 74 patients were evaluable for clinical/microbiological efficacy, and PK/PD analysis. The median age of the 74 patients was 67 years (IQR, 55–78) (Table 1). Cardiovascular diseases were the most common comorbidities, followed by diabetes mellitus. Among the 11 patients with solid tumors, the incidence was significantly higher in the treatment failure group than in the success group. For the enrolled patients with cUTIs, their baseline creatinine clearance is 56.2 (IQR, 36.1–96.0) mL/min. Among them, 20 patients had co-infections with other Gram-positive bacteria (Supplementary Table S1), primarily involving bloodstream and pulmonary infections. In the overall cohort, 46.2% of patients had a vascular catheter, 24.3% had a urinary catheter, and 32.4% were admitted to the intensive care unit; none of these exposures were significantly associated with treatment outcomes (P > 0.05). A total of 39 patients (52.7%) received concurrent antibacterial agents (Table 1), mainly including piperacillin/tazobactam, ceftazidime, cefoperazone/sulbactam, and meropenem, for confirmed or suspected Gram-negative bacterial infections, as well as concomitant antifungals for fungal infections. Only four patients also received fluoroquinolones, and two patients received fosfomycin (which has activity against Gram-positive pathogens). Although these agents could potentially confound the assessment of vancomycin efficacy, their impact was likely limited; however, confounding cannot be fully excluded, and larger studies are needed.

**TABLE 1:** Baseline clinical characteristics of patients in terms of vancomycin treatment outcomes.

Characteristics	Total (N = 74)	Treatment success (N = 64)	Treatment failure (N = 10)	*P* value
Demographics				
Sex (male), n (%)	37 (50.0)	30 (46.9)	7 (70.0)	0.308
Age (years), median (IQR)	67 (55–78)	66 (52–79)	67 (62–77)	0.496
Body weight (kg), median (IQR)	63 (58–70)	65 (58–70)	60 (55–70)	0.174
BMI (kg/m^2^), median (IQR)	23.23 (20.76–25.32)	22.88 (20.76–24.03)	23.34 (20.76–25.34)	0.556
Comorbidities				
Cardiovascular disease, n (%)	40 (54.1)	36 (56.3)	4 (40.0)	0.497
Diabetes mellitus, n (%)	25 (33.8)	22 (34.4)	3 (30.0)	>0.999
Shock, n (%)	14 (18.9)	12 (18.8)	2 (20.0)	>0.999
Solid tumor, n (%)	11 (14.9)	6 (9.4)	5 (50.0)	0.005*
Prior surgery, n (%)	26 (35.1)	21 (32.8)	5 (50.0)	0.307
Exposures				
Vascular catheter, n (%)	32 (46.2)	26 (40.6)	6 (60.0)	0.313
Urinary catheter, n (%)	18 (24.3)	15 (23.4)	3 (30.0)	0.697
ICU admission, n (%)	24 (32.4)	20 (31.3)	4 (40.0)	0.719
Co-infections*	20 (27.0)	16 (25.0)	4 (40.0)	0.444
Pneumonia, n (%)	7 (9.5)	6 (9.4)	1 (10.0)	>0.999
BSI, n (%)	9 (12.2)	8 (12.5)	1 (10.0)	>0.999
cIAI, n (%)	4 (5.4)	3 (4.7)	1 (10.0)	0.448
SSTI, n (%)	2 (2.7)	1 (1.6)	1 (10.0)	0.254
Baseline serum creatinine (μmol/L), median (IQR)	87.8 (60.0–132.1)	88.0 (62.4–149.3)	74.1 (52.0–119.0)	0.496
Baseline creatinine clearance (mL/min)^a^, median (IQR)	56.2 (36.1–96.0)	56.2 (35.8–96.0)	58.6 (49.0–98.0)	0.927
Pathogens				
*E. faecalis*, n (%)	30 (40.5)	28 (43.8)	2 (20.0)	0.187
*E. faecium*, n (%)	24 (32.4)	19 (29.7)	5 (50.0)	0.277
*S. aureus*, n (%)	9 (12.2)	6 (9.4)	3 (30.0)	0.097
*Streptococcus*, n (%)	10 (13.5)	10 (15.6)	0 (0)	0.339
MIC≥2 mg/L, n (%)	7 (9.5)	5 (7.8)	2 (20.0)	0.238
Vancomycin therapy				
Duration (days), median (IQR)	7 (6–11)	7 (6–11)	9 (7–12)	0.387
Initial daily dose (g/d), median (IQR)	1.0 (1.0–2.0)	1.0 (1.0–2.0)	1.5 (1.0–2.0)	0.244
Dose adjustment, n (%)	15 (20.3)	13 (20.3)	2 (20.0)	>0.999
Adjusted daily dose (g/d), median (IQR)	1.0 (1.0–2.0)	1.0 (1.0–1.25)	1.25 (1.0–2.0)	0.103
Concurrent antibacterial agents, n (%)	39 (52.7)	32 (50.0)	7 (70.0)	0.316
Concurrent penicillins^b^, n (%)	9 (12.2)	7 (10.9)	2 (20.0)	0.600
Concurrent cephalosporins^c^, n (%)	14 (18.9)	12 (18.8)	2 (20.0)	>0.999
Concurrent carbapenems^d^, n (%)	12 (16.2)	10 (15.6)	2 (20.0)	0.661
Concurrent fosfomycin, n (%)	2 (2.7)	2 (3.1)	0 (0)	>0.999
Concurrent antifungals, n (%)	10 (13.5)	8 (12.5)	2 (20.0)	0.617
Concurrent quinolones, n (%)	4 (5.4)	3 (4.7)	1 (10.0)	0.448
Concurrent other antibacterial agents^e^, n (%)	7 (9.5)	6 (9.4)	1 (10.0)	>0.999

* *P* < 0.05.

^a^ Creatinine clearance was calculated by the Cockcroft-Gault formula. * Four patients had infections at three sites each. Two patients with central nervous system (CNS) infection and cUTIs, achieved treatment success; see Supplementary Table S1 for details.

BMI, body mass index; ICU, intensive care unit; BSI, bloodstream infection; cIAI, complicated intra-abdominal infection.

^b^ The main penicillins included two β-lactam/β-lactamase inhibitors: piperacillin/tazobactam (7/74, 9.5%) and ampicillin/sulbactam (2/74, 2.7%).

^c^ The main cephalosporins included ceftazidime (6/74, 8.1%), cefoperazone/sulbactam (5/74, 6.8%), and ceftriaxone (2/74, 2.7%).

^d^ The main carbapenems included meropenem (11/74, 14.9%) and imipenem (1/74, 1.4%).

^e^ Other concomitant antibacterial agents included metronidazole, doxycycline, and rifampin.

### 3.2 Microbiological data and PK/PD analysis

A total of 74 strains of Gram-positive uropathogens were isolated from urine cultures, including *E*. *faecalis* (30/74), *Enterococcus faecium* (25/74), *Streptococcus* (10/74), and *S*. *aureus* (9/74). Among the patients with *S. aureus* infections, 8 cases were identified as MRSA infections. Specifically, MRSA was also detected in sputum samples from two patients with pneumonia and in tissue secretion samples from one patient with SSTI (Supplementary Table S1). Among patients with concurrent bloodstream infections, four were diagnosed with bloodstream infections caused by methicillin-resistant coagulase-negative staphylococci alongside cUTIs due to *Enterococcus* spp. The other five patients had concordant pathogens isolated from both bloodstream and urine cultures, including 3 cases of *E*. *faecalis*, one case of methicillin-sensitive *S*. *aureus* (MSSA), and one case of *Streptococcus agalactiae*. Based on the temporal sequence of infection and clinical judgment, two of these cases, one attributed to *E. faecalis* and the other to *S. agalactiae*, were considered likely to represent bloodstream infections secondary to cUTIs.

**FIGURE 2:**
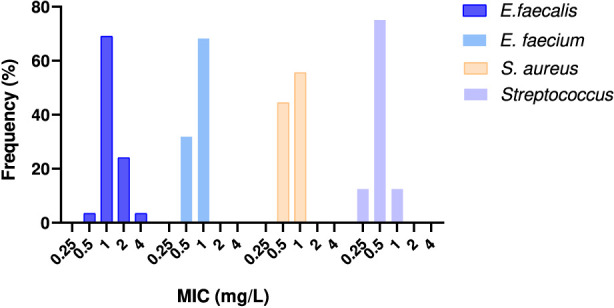
Minimum inhibitory concentration distribution of vancomycin for target pathogens. MIC, minimal inhibitory concentration.

Higher proportions of *S. aureus* and *E. faecium* were observed in the treatment failure group, although the differences were not statistically significant (Table 1). The MIC distribution of vancomycin against different uropathogens is illustrated in Figure 2. For *E. faecalis*, the vancomycin MIC_90_ is 2 mg/L, with one strain having an MIC of 4 mg/L. For other pathogens, the MIC_90_ values are all 1 mg/L. No resistant organisms were found, indicating that the uropathogens currently collected exhibit good sensitivity to vancomycin, and no emergence of resistance or MIC increase was observed during the treatment period.

**FIGURE 3:**
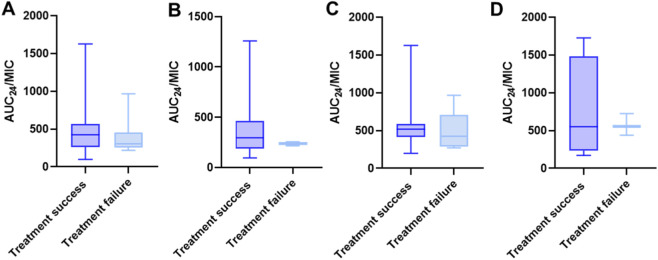
Comparison of pharmacokinetic/pharmacodynamic indices between vancomycin treatment success and failure groups across target pathogens. **(A)** *Enterococcus* spp; **(B)** *E. faecalis*; **(C)** *E. faecium*; **(D)** *S. aureus*. AUC_24_/MIC: 24-h are under the concentration-time curve to minimal inhibitory concentration.

For the patients with cUTIs, the median duration of vancomycin therapy was 7 (IQR, 6–11) days. The median initial daily dose of vancomycin was 1.0 (IQR, 1.0–2.0) g/d, given in divided doses every 12 h or as a once-daily regimen. Among the 74 patients, the median serum C_min_ of vancomycin was 9.22 (IQR, 4.36–14.33) mg/L and peak concentration (C_max_) of 22.56 (IQR, 17.15–30.70) mg/L. The median AUC_24_ and AUC_24_/MIC values were 455 (IQR, 277–537) mgh/L and 455 (IQR, 268–627), respectively. Vancomycin C_min_ segmentations (<10, 10–15, >15 mg/L) and AUC_24_/MIC segmentations (<400, 400–600, >600) were analyzed respectively in terms of treatment outcomes (success or failure), but no significant association was found (Table 2). The proportion of patients within the effective C_min_ range (10–15 mg/L) and AUC_24_/MIC range (400–600) was relatively low, with the majority of patients falling within subtherapeutic concentrations (<10 mg/L for C_min_) and AUC_24_/MIC values (<400). The AUC_24_ and AUC_24_/MIC were further compared in terms of vancomycin treatment outcome by different pathogens (Figure 3). For *Enterococcus* spp., although no statistically significant difference was observed, the treatment success group exhibited higher trends in AUC_24_/MIC compared to the failure group.

**TABLE 2:** Association between pharmacokinetic/pharmacodynamic indices and vancomycin treatment outcomes.

Characteristics	Total (N = 74)	Treatment success (N = 64)	Treatment failure (N = 10)	*P* value
C_max_ (mg/L), median (IQR)	22.56 (17.15–30.70)	22.08 (17.24–29.67)	24.01 (13.57–32.75)	0.469
C_min_ (mg/L), median (IQR)	9.22 (4.36–14.33)	9.04 (3.71–13.63)	10.10 (5.88–23.59)	0.160
<10 mg/L, n (%)	39 (52.9)	36 (56.3)	3 (30.0)	0.176
10–15 mg/L, n (%)	22 (29.7)	19 (29.7)	3 (30.0)	>0.999
>15 mg/L, n (%)	13 (17.6)	9 (14.1)	4 (40.0)	0.067
AUC_24_, mg·h/L, median (IQR)	455 (277–537)	456 (265–537)	447 (303–510)	0.239
AUC_24_/MIC, median (IQR)	455 (268–627)	465 (263–639)	435 (269–554)	0.174
<400, n (%)	28 (37.8)	24 (37.5)	4 (40.0)	>0.999
400–600, n (%)	27 (36.5)	23 (35.9)	4 (40.0)	>0.999
>600, n (%)	19 (25.7)	17 (26.6)	2 (20.0)	>0.999

C_max_, peak concentration; C_min_, trough concentration; AUC_24_, 24-h area under concentration-time curve; MIC, minimal inhibitory concentration.

Among the enrolled patients, 15 had their vancomycin dosages adjusted (Table 1). However, there was no significant difference in the proportion of dosage adjustments between the treatment failure group and the success group. For the two patients with concomitant bloodstream infections, one had their vancomycin dosage reduced based on TDM results, while the other did not undergo any dosage adjustment. Both patients ultimately achieved treatment success and bacterial clearance.

### 3.3 Safety analysis

Three patients identified with vancomycin-related nephrotoxicity. All C_min_ in the three patients were collected within 30 min prior to the next dose at steady-state. In one case, a patient with a tumor had the SCr increase by ≥ 2-fold compared to baseline, within 4 days of dosing. The patient received a dose of 0.5 g every 12 h (q12 h) and achieved a steady-state C_min_ of up to 31.54 mg/L (day 7). However, upon discontinuation of vancomycin, the patient’s renal function showed improvement. In the second case, SCr values increased by 29 μmol/L on the second day of treatment. The patient was initially dosed at 0.5 g q8h, and a serum TDM was performed on day 4 of treatment, with a C_min_ of 23.88 mg/L and a C_max_ of up to 36.59 mg/L. After discontinuing the drug on day 5 of treatment without any dosage adjustments, the SCr value remained high, and there was no subsequent follow-up. The third patient initially received vancomycin 0.5 g q12 h. By day 4, the steady-state C_min_ was 15.33 mg/L, accompanied by a rise in SCr from 101.6 to 140.6 μmol/L. The dose was then reduced to 0.5 g daily. Subsequent TDM showed C_min_ between 7.41 and 12.27 mg/L, yet SCr continued to increase: 169.2 μmol/L (day 11); 172.2 μmol/L (day17). On day 19, a trough sample revealed a C_min_ of 21.93 mg/L, and SCr reached 227.9 μmol/L. As bacterial clearance had been achieved and SCr had doubled from baseline, vancomycin was discontinued. On day 21, SCr rose to 277.5 μmol/L and continuous renal replacement therapy (CRRT) was initiated. The patient ultimately died from multiple organ failure on day 26 of therapy.

### 3.4 Efficacy and risk factors

Overall, vancomycin treatment was successful in 64 (86.5%) patients and failure in 10 (13.5%) patients. Exploratory analyses suggested that solid tumor and *S. aureus* infection were associated with treatment failure (Table 3). However, due to the small number of events and wide confidence intervals, these findings should be validated in larger cohorts.

**TABLE 3:** Exploratory multivariable logistic analysis on vancomycin treatment outcomes.

Variable^a^	Univariate analysis OR (95% CI)	Multivariate analysis	
		aOR (95% CI)	*P* value
Solid tumor	9.667 (2.162, 43.223)	13.137 (2.522, 68.865)	0.002
*S. arues*	4.143 (0.843, 20.364)	6.857 (1.083, 43.408)	0.041
C_min_ >15 mg/L	4.074 (0.957, 17.337)	​	​

^a^ OR, odds ratio; CI, confidence interval; aOR, adjusted odds ratio.

Among the 11 patients with solid tumor, only 3 (27.3%) patients achieved either an initial C_min_ of 10–15 mg/L or an AUC_24_/MIC of 400–600 (Supplementary Table S2). Of note, among the enrolled solid tumor patients, 10 had *Enterococcus* infections (MIC_90_ = 4 mg/L). Of the 6 cases infected with *E*. *faecalis*, the vancomycin MIC for isolates from two patients reached 2 mg/L, and one isolate showed an MIC as high as 4 mg/L.

Compared to *Enterococcus*, cUTIs caused by *S. aureus* are less common, with generally lower MIC values and higher AUC_24_/MIC ratios (554 vs. 355) than those observed in solid tumor patients. Although the overall rate of achieving the target AUC_24_/MIC was not high, 77.7% of patients still attained an AUC_24_/MIC >400. Considering the complexity of *S. aureus* infections, we conducted heteroresistance testing and whole-genome sequencing on eight urinary isolates as previously described (Fan et al., 2022) to determine clonal complexes (CC). All strains were identified as vancomycin-susceptible *S. aureus* (VSSA), of which six belonged to the CC5 clone: one was sequence type 5 (ST5) and five were ST764 (Table 4). These findings suggest that treatment failure was not attributable to vancomycin resistance. Given the small number of *S. aureus* isolates, these results are exploratory and hypothesis-generating. Further studies are needed to investigate the potential impact of CC5-type *S. aureus* on therapeutic outcomes in cUTIs.

**TABLE 4:** Molecular typing of urinary-derived *Staphylococcus aureus*.

Strains^a^	Classification according to methicillin resistance	hVISA screening	Sequence type (ST)	Clone complex (CC)	Efficacy
S1	MRSA	VSSA	ST5	CC5	Success
S2	MRSA	VSSA	ST1	CC1	Success
S3	MRSA	VSSA	ST764	CC5	Failure
S4	MRSA	VSSA	ST764	CC5	Success
S5	MRSA	VSSA	ST764	CC5	Success
S6	MRSA	VSSA	ST764	CC5	Failure
S7	MRSA	VSSA	ST764	CC5	Failure
S8	MSSA	VSSA	New ST	CC88	Success

MRSA: methicillin-resistant *Staphylococcus aureus;* MSSA: methicillin-susceptible *S. aureus*; hVISA: heteroresistant vancomycin-intermediate *S. aureus*; VSSA, vancomycin-susceptible *S. aureus*.

^a^ One strain of bacteria failed to be sent for gene sequencing in a timely manner.

## 4 Discussion

Vancomycin exhibits a high urinary excretion rate (80%–90%) and achieves a high concentration in urine. The necessity of serum vancomycin TDM in patients with cUTIs remains debated. Despite the low probability of achieving effective C_min_ and AUC_24_/MIC in patients with cUTIs, vancomycin demonstrates good clinical efficacy.

To investigate this discrepancy, we explored the potential link between vancomycin PK/PD indices and treatment outcomes in patients with cUTIs. Although no significant correlation was found between these PK/PD indices and clinical efficacy, a high clinical efficacy and a microbiological eradication rate of 86.5% were observed, with a median AUC_24_/MIC of approximately 400. It is important to recognize that the conventional AUC_24_/MIC target range of 400–600 was primarily established for MRSA infections, where the lower limit (400) is associated with efficacy and the upper limit (600) is mainly derived from nephrotoxicity risk as a safety cutoff. Extrapolating this range to other Gram-positive pathogens, especially in the context of cUTIs, remains uncertain. Currently, PK/PD target values for vancomycin in the treatment of enterococcal infections are primarily based on studies of bloodstream infections, with three studies reporting a correlation between AUC_24_/MIC and efficacy at values of 389 and 414.3 (Jumah et al., 2018; Komatsu et al., 2023; Tochikura et al., 2024), respectively, both approximating 400. However, other studies have found no correlation between AUC_24_/MIC and efficacy (Sasano and Hanada, 2023). For *Streptococcus* spp., specific PK/PD targets are lacking.

Most contemporary international guidelines (Kranz et al., 2024; Nelson et al., 2024; Trautner et al., 2025) for urinary tract infection focus primarily on empiric therapy for Gram-negative pathogens. The 2025 Infectious Diseases Society of America (IDSA) guideline recommends a four-step approach (severity, resistance risk, patient factors, antibiogram) for selecting empiric antibiotics, with preferred agents being third-/fourth-generation cephalosporins, piperacillin-tazobactam, carbapenems, or fluoroquinolones. Vancomycin is not listed as a first-line empiric option; it is mentioned only as an agent that may be considered in septic patients when the diagnosis is unclear or when enterococcal or MRSA infection is suspected. Thus, vancomycin is positioned as a targeted therapy for documented or highly suspected MRSA or ampicillin-resistant *Enterococcus*, rather than as routine empiric coverage. This aligns with the other guidelines, which also reserve vancomycin for targeted use against resistant Gram-positive pathogens (He et al., 2020; Rybak et al., 2020; Brown et al., 2021).

In our cohort of cUTIs, *Enterococcus* spp. (31/47) were the predominant pathogens and remained relatively susceptible to vancomycin. Among enterococcal cUTIs, although case numbers were limited, higher AUC_24_ and AUC_24_/MIC values were observed in the treatment success group, suggesting potential clinical value for serum TDM. In our study, *E*. *faecalis* was the main uropathogen, with MIC_50_ and MIC_90_ values of 1 mg/L and 2 mg/L, respectively; one isolate had an MIC of 4 mg/L. For *E*. *faecalis* with MICs of 2–4 mg/L, achieving an AUC_24_/MIC ≥400 with standard dosing is challenging. Therefore, low serum AUC_24_/MIC alone did not predict treatment failure, because urinary vancomycin concentrations are much higher and likely drive efficacy.

The presence of a solid tumor was noted as a potential risk factor in exploratory analysis. Among these patients, the primary pathogen was *Enterococcus*, and treatment in this group was characterized by low attainment rates of both the C_min_ and AUC_24_/MIC targets. Saeed et al. (Alqahtani et al., 2020) reported that patients with cancer had a significantly higher vancomycin clearance than noncancer patients, thus vancomycin levels were significantly lower in patients with cancer.

In addition to host factors, the pathogen profile itself influenced outcomes. *S. aureus* infection, particularly MRSA, was another risk factor for treatment failure in the exploratory analysis. While an AUC_24_/MIC ratio of 400–600 is recommended for all serious MRSA infections, and MRSA has emerged as a significant cause of hospital-acquired UTIs. Although its overall prevalence in urinary isolates is low (approximately 2%–10% globally) (Kumar et al., 2025), MRSA cUTIs can lead to severe, life-threatening clinical deterioration and therefore warrant heightened vigilance. In this study, we included nine cases of *S. aureus*-associated cUTIs with MICs ≤1 mg/L and no detected heteroresistance. This suggests that mechanisms beyond susceptibility, such as enhanced virulence, may be at play. A recent genomic study of long-term catheter users reported persistent MRSA (ST5/CC5) despite antibiotic therapy and catheter exchanges (Duran Ramirez et al., 2026), supporting the clinical relevance of this lineage. Study by Paudel et al. (Paudel et al., 2021) reports that human urine can alter the virulence and transcriptome of MRSA, and the predominant CC5 clone in our isolates may represent such a virulent lineage.

Of particular clinical concern, we also found that the bloodstream infections in two of the patients with cUTIs might have originated from their cUTIs. Hospital-acquired urinary tract-associated bloodstream infections have a low incidence but a high mortality rate. Among adult inpatients admitted to four Veterans Affairs hospitals in the United States, the mortality rate for patients with hospital-acquired urinary tract-associated bloodstream infections caused by *Staphylococcus* was 25.8%, and the mortality rate for patients with infections caused by *Enterococcus* was 20.7% (Patel et al., 2018). Therefore, although the incidence is low, urinary-source bloodstream infections still warrant increased clinical attention.

Consequently, a key practical question arises: should TDM be performed in serum or urine during vancomycin treatment for cUTIs? One Study has measured the trough serum and urine concentrations of vancomycin post-treatment, with mean values of 13.13 ± 1.34 mg/L and 7.79 ± 1.23 mg/L, respectively, showing a positive linear correlation (Shokouhi et al., 2017). As vancomycin is primarily excreted within 6 h post-dose, C_min_ monitoring alone is insufficient to fully represent urine vancomycin levels. We explored to collect 24-h urine samples from three patients with cUTIs, revealing average concentrations exceeding our detection limit (100 mg/L). Thus, urine vancomycin levels are relatively high post-administration, and bacterial clearance remains promising despite some *E*. *faecalis* strains having an MIC of 2–4 mg/L. Currently, some studies have also demonstrated the feasibility of using urine concentration for TDM (Oshima et al., 2025), including the ability to predict the AUC_24_. However, urine concentration is influenced by various factors such as fluid load and water intake, so the timing of monitoring and its feasibility require further investigation.

**FIGURE 4:**
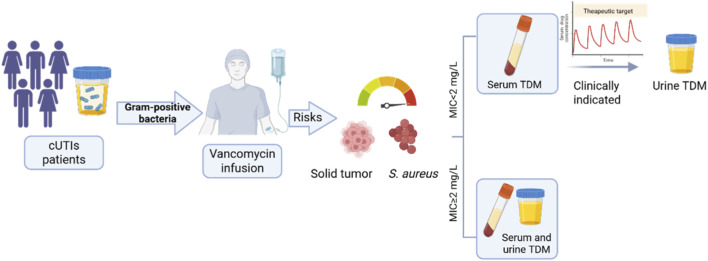
Strategies and pathways for optimizing vancomycin therapy through TDM in patients with cUTIs. This figure was created with BioRender.com, with a commercial academic license for publication use.

Based on this study and literature reports, we recommend a stratified management approach for patients with cUTIs treated with vancomycin (Figure 4). The risk of treatment failure should be assessed alongside nephrotoxicity, and therapy should be guided by the pathogen’s MIC: For an MIC <2 mg/L, serum TDM should be used to achieve target AUC_24_/MIC ratios and improve efficacy. For an MIC ≥2 mg/L, serum AUC_24_/MIC targets are often unachievable, and urinary TDM may offer a valuable alternative. In patients demonstrating subtherapeutic serum concentrations and a low target attainment probability based on serum TDM, the addition of urinary monitoring is advisable when clinically indicated.

This study has several limitations. First, the small sample size precluded determination of cUTI-specific vancomycin PK/PD targets, and the risk factors (solid tumor and urinary *S. aureus*) for treatment failure require validation in larger cohorts. Second, the conventional PK/PD target (AUC_24_/MIC 400–600) was derived from MRSA infections and may not be directly applicable to other Gram-positive pathogens in cUTIs. Third, the KDIGO urine output criterion was defined but not collected in our forms, so we could not assess AKI by urine output. Fourth, we did not assess sarcopenia or weight loss, which can affect serum creatinine-based renal function estimates (Li et al., 2025), and cystatin C (a more accurate marker of renal function) was not measured.

## 5 Conclusion

In this cohort of patients with Gram-positive cUTIs, vancomycin was effective in *Enterococcus*-dominant infections and showed a modest response in a small number of MRSA cases, despite low PK/PD target attainment. Most patients received concomitant antibiotics targeting Gram-negative bacteria or fungi. The presence of solid tumor and *S. aureus* infection showed exploratory associations with increased risk of treatment failure. Serum C_min_ and AUC_24_/MIC were not significantly associated with treatment outcome. Therefore, while serum PK/PD-guided TDM is not supported for efficacy prediction, it remains valuable for safety monitoring and individualized dosing to optimize exposure and reduce adverse reactions. Further validation with larger patient cohorts is required to confirm these findings.

## Data Availability

The original contributions presented in the study are included in the article/Supplementary Material, further inquiries can be directed to the corresponding author.
