# Supplementary material for: Limited association between serum vancomycin pharmacokinetic/ pharmacodynamic indices and clinical outcomes in gram-positive complicated urinary tract infections
Source: Front Pharmacol. 2026 Jul 16;17:1772367. doi: 10.3389/fphar.2026.1772367 (PMC13421436; doi:10.3389/fphar.2026.1772367)
Supplement: Supplementary file 1 [file Supplementaryfile1.pdf]

Table S1. Co-infection in patients with cUTIs

Patients	Urinary pathogen	Co-infection site	Co-infected organism	Concordance with urine culture	concurrent antibacterial agents	Treatment outcome
A-HS-048	*E. faecalis*	Pneumonia	*S. aureus*	N	Doxycycline	Y
A-ZS-046	*E. faecalis*	BSI	*E. faecalis*	Y	/	Y
A-ZS-044	*E. faecium*	cIAI	*E. faecium*	Y	Meropenem	N
A-1Y-037	*E. faecium*	Pneumonia	*S. aureus*	N	/	Y
A-PR-012	*S. aureus**	Pneumonia	*S. aureus*	Y	Ciprofloxacin , Metronidazole , Fluconazole, Moxifloxacin	N
PR-011	*S. aureus**	SSTI	*S. aureus*	Y	Cefotaxime	N
A-1Y-038	*S. aureus**	Pneumonia	*S. aureus*	Y	Piperacillin/tazobactam	Y
RJ-031	*S. agalactiae*	BSI	*S. agalactiae*	Y	Meropenem	Y
75	*E. faecalis*	BSI, cIAI	*E. faecalis*	Y	Imipenem/cilastatin	Y
240	*S. aureus***	BSI, SSTI	*S. aureus*	Y	Imipenem/cilastatin	Y
272	*E. faecalis*	Pneumonia	*S. aureus*	N	Ampicillin/sulbactam	Y
284	*E. faecalis*	BSI	*S. capitis*	N	Piperacillin/tazobactam , Fluconazole	Y
285	*E. faecalis*	Pneumonia, cIAI	*E. faecalis*	Y	Cefoperazone, Metronidazole, Sultamicillin	Y
359	*E. faecium*	BSI	S. hominis	N	Ceftazidime	Y
426	*E. faecium*	Pneumonia	*S. aureus*	N	Meropenem, Cefoperazone/sulbactam	Y
449	*E. faecalis*	CNS	*S. haemolyticus*	N	/	Y
933	*E. faecalis*	BSI	*E. faecalis*	Y	Caspofungin, Meropenem, Ceftazidime, Cefepime	Y
936	*E. faecalis*	BSI, cIAI	*S. hominis*, *E. faecalis*	Y	Cefoperazone/sulbactam, Meropenem	Y
157	*E. faecalis*	CNS	S. epidermidis	N	Levofloxacin, Piperacillin/tazobactam, Fosfomycin	Y
275	*E. faecium*	BSI	S. hominis	N	/	N

*MRSA: methicillin- susceptible *Staphylococcus aureus*

** MSSA: methicillin-resistant *Staphylococcus aureus*

Table S2. Vancomycin PK/PD indices in cUTIs patients with solid tumors or *S. aureus*

Characteristics	Patient with solid tumors (N=11)	Patient with *S. aureus* (N=9)
C_{min} (mg/L)	13.15 (8.81, 18.82)	14.9 (5.88, 18.56)
< 10	3 (27.3)	3 (33.3)
10 – 15	3 (27.3)	2 (22.2)
>15	5 (45.5)	4 (44.4)
AUC_{24}/MIC	355 (269, 500)	554 (444, 1483)
< 400	6 (54.5)	2 (22.2)
400 – 600	3 (27.3)	3 (33.3)
> 600	2 (18.2)	4 (44.4)

Supplementary file: Vancomycin−induced nephrotoxicity was diagnosis and assessment

Vancomycin−induced nephrotoxicity was diagnosed by attending clinicians based on predefined criteria in the study protocol. Vancomycin-related nephrotoxicity indicates the occurrence of acute kidney injury (AKI). According to the KDIGO criteria (Khwaja, 2012), AKI includes any of the following conditions: (a) an increase in serum creatinine (SCr) by ≥26.5 μmol/L within 48 hours; (b) a known or presumed increase in SCr by 1.5 times the baseline value within the past 7 days; (c) urine output less than 0.5 mL/kg/h within 6 to 12 hours.

A causality assessment was performed using the following objective criteria, adapted from standard pharmacological evaluation:

(1) Definite: Clear temporal relationship, no alternative explanation, improvement after withdrawal, and positive rechallenge (if feasible).

(2) Probable: Clear temporal relationship, no better alternative explanation, and improvement after withdrawal.

(3) Possible: Reasonable temporal relationship, other explanations possible, withdrawal information unclear or unavailable.

(4) Unlikely: Another cause more likely; no improvement after withdrawal or information unclear.

(5) Unrelated: No temporal relationship or clear alternative cause.

Cases categorized as definite, probable, or possible were considered related to vancomycin. Other potential causes of acute kidney injury (e.g., other nephrotoxic drugs) were excluded by the clinical team before assigning causality.
